# Efficient and simple protocol for mechanical isolation of foreskin-derived primary human dermal fibroblasts for replicative senescence studies

**DOI:** 10.1186/s13104-025-07373-2

**Published:** 2025-07-18

**Authors:** Astrid Feinisa Khairani, Nayla Majeda Alfarafisa, Yoan Chou, Widad Aghnia Shalannandia, Maiko Ikezawa, Achadiyani Achadiyani, Nur Atik

**Affiliations:** 1 Department of Biomedical Sciences, Faculty of Medicine, Universitas Padjadjaran, Bandung, West Java Indonesia; 2 Graduate School of Master Program in Anti Aging and Aesthetic Medicine, Faculty of Medicine, Universitas Padjadjaran, Bandung, West Java Indonesia; 3 Biomedical Science Master Program, Faculty of Medicine, Universitas Padjadjaran, Bandung, West Java Indonesia; 4 Department of Anatomy and Cell Biology, Graduate School of Medicine, Gunma University, Gunma, Japan

**Keywords:** Dermal fibroblast, Fibroblast-derived human, Mechanical isolation, Primary cell culture, Replicative senescence

## Abstract

**Background:**

An efficient and reproducible protocol was developed for the mechanical isolation of human dermal fibroblasts (HDFs) from the foreskin, providing a practical alternative to enzymatic methods. This protocol could be easily performed with limited resources by reducing the need for expensive reagents and complex procedures. Viable cells were successfully subcultured repeatedly for cellular senescence studies, with reproducibility ensured through a detailed description.

**Results:**

This mechanical isolation method successfully generated HDFs with elongated spindle-shaped morphological characteristics that expressed the ACTIN protein, VIMENTIN protein, and *SERPINH1* gene. HDFs survived through passage 8 (P8) during repeated subculturing.

**Conclusions:**

The mechanical isolation protocol efficiently yields primary HDFs from the foreskin and supports replicative senescence up to passage 8 (P8). It can be applied to intrinsic skin aging studies, particularly in resource-limited settings.

**Supplementary Information:**

The online version contains supplementary material available at 10.1186/s13104-025-07373-2.

## Introduction

Fibroblast primary culture can be accomplished through enzymatic or mechanical isolation [1–5]. Enzymatic solution, which rely on digestion enzymes such as trypsin or collagenase, present several challenges, including cell viability loss due to enzyme sensitivity, increased cost, and procurement difficulties in regions with limited access to laboratory reagents, which further contribute to expenses, particularly in middle-income countries that depend on imports [6]. The additional cost resulted from the need for extra digestion enzymes in enzymatic isolation and the requirement of a larger volume of complete medium to halt enzymatic digestion, which contained antimicrobials and serum [6]. Additionally, given the heightened sensitivity of primary cell cultures, the inappropriate use of enzyme concentrations may lead to low cell viability, failing at the initial stage of cell culture cultivation [6, 7]. A study demonstrated that trypsin solution on tissue slurry caused cell lysis and clump formation.

In contrast, mechanical isolation eliminates the need for enzymatic digestion, offering a cost-effective and accessible alternative. Previous studies have demonstrated that mechanical isolation preserves cell viability, reduces apoptosis, and prevents necrosis, making it a viable method for primary fibroblast culture [6]. Consistent with these findings, our study observed that trypsin initially failed to promote cell growth during primary culture. However, excluding trypsin in the initial stage allowed for successful cell growth, with its use being limited to subculturing.

Hence, we have optimized a more accessible and cost-efficient method—specifically, a mechanical isolation protocol —to reduce expenses associated with the initial stages of cell culture. In this protocol, foreskin was selected due to its availability as biological waste from circumcision procedures, making it an ethically and practically viable source of human dermal fibroblasts (HDFs). Globally, male circumcision rates is performed at a high prevalence (36.7–38.7%), with near-universal rates (99.9%) in predominantly Muslim or Jewish populations, ensuring consistent sample availability [8]. Furthermore, in fibroblast characterization, ACTIN, VIMENTIN, and SERPINH1 were selected due to their roles in maintaining fibroblasts’ structural integrity and function, as they are key cytoskeletal components and important in collagen stability. Previous studies have identified these markers in human fibroblasts, highlighting their relevance for fibroblast characterization [1]. 

HDFs, predominant in the dermis, are widely used in aging studies. Cellular aging can be induced through various methods, including repeated subculturing, which leads to replicative senescence [9, 10]. This model reflects natural aging and provides a valuable alternative to other aging models, such as those induced by ultraviolet radiation, oxidative stress, or high glucose levels [11]. Therefore, we optimized a repeated subculturing method until HDFs reached passage (P)8 for replicative senescence studies.

## Materials and methods

All biological materials procedures should be conducted in a standardized laboratory with Biosafety Level (BSL) 1 or 2. Additionally, all solutions and instruments must be sterile to ensure experimental integrity and biosafety compliance. Foreskin samples from three (*n* = 3) healthy male donors (< 10 years) were collected with informed consent. Exclusion criteria included circumcision contraindications, preputial abnormalities, and systemic skin disorders. Ethical clearance and informed consent are mandatory for studies involving human material. This research adhered to ethical standards set by Universitas Padjadjaran, with ethics approval number 1115/UN6.KEP/EC/2023.

### Materials

The reagents and solutions needed for this mechanical isolation protocol are transport medium, Complete medium Dulbecco’s Modified Eagle (DMEM-C), Dulbecco’s Phosphate Buffered Saline (DPBS) 1X, and Trypsin 0.05% for subculture. The detailed recipes are mentioned in Additional File 1, Table S1. All the solutions were filtered using Syringe-Filter 0.22 μm, PES membrane (TPP Spritzen, 99722). DMEM-C was used as a standard fibroblast culture medium. Transport medium, DMEM-C, and DPBS-1X were stored at 4^o^C. Trypsin 0.05% was stored at -20^0^C. Before initiating the protocol, all reagents and solutions were pre-warmed using a water bath set at 37 °C. Mechanical isolation protocol requires a sterile surgical set, including autoclaved forceps, scissors, and a surgical blade.

### Methods

#### HDFs mechanical isolation from foreskin

After obtaining the sample, the sample was transported to the laboratory using a cold transport medium equipped with an ice box and ice gel. The sample was decontaminated with povidone-iodine for 15 s, 70% alcohol for 5 s, and DPBS-1X three times in a sterile petri dish. In a DPBS-1X, the sample was cut into two parts for easier cleaning of the inner sections, and the foreskin tip (at the urethral meatus section) was discarded, as this area typically contains a high amount of difficult-to-clean bacterial residue, leading to potential contamination.

The HDFs were mechanically isolated by separating the adipose layer with sterile scissors or a blade while the sample was in a petri dish containing DMEM-C and delicately scraping the epidermis layer with a blade while the sample was in a petri dish containing DMEM-C. The adipose and epidermis layers were discarded. The dermis (white tissue) was cut into small fragments (explant) using scissors or a blade, aiming for sizes around 1–2 mm.

Explants were seeded into 25 cm² (T-25) flasks (Thermo Fisher Scientific, 156367), with six explants per flask. The explants were left for 15 min without a medium to ensure optimal adhesion, after which 2–3 ml of DMEM-C was gently added to avoid tissue displacement. HDFs were incubated at 37 °C with 5% CO₂.

**Fig. 1:**
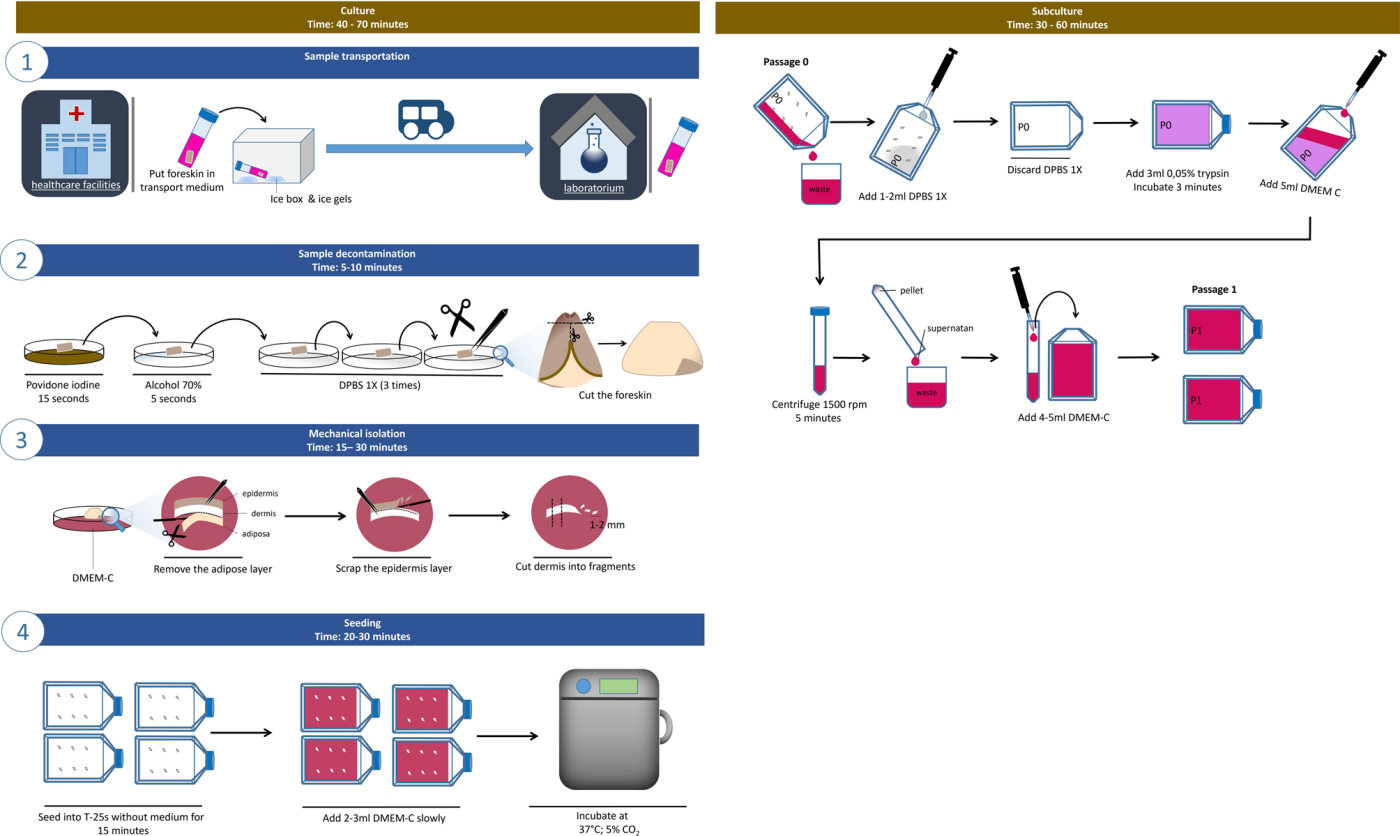
Protocol for mechanical isolation and subculture of HDFs

Cultures were observed every 1–3 days to indicate signs of contamination, and the DMEM-C medium was replaced every 2–5 days or before drying out (Fig. 1). During the initial cultivation phase, the FBS concentration in DMEM-C was increased to 12–15% until visible cell growth was observed, then adjusted back to 10% after stable growth. HDF characterization to identify spindle-shaped cells was conducted with a phase contrast inverted microscope (Olympus CK40 Phase Contrast Microscope, 40x magnification) and documented using ToupView software.

#### HDFs subculture to induce replicative senescence

HDFs were subcultured at 80–90% confluence using enzymatic methods. Before initiating the subculture, the previous DMEM-C medium was removed, and cells were rinsed with DPBS-1X and discarded. Then, 3 ml of 0.05% trypsin was added, followed by a 3–5-minute incubation. After confirming HDFs detachment under a microscope, trypsin activity was halted by adding 5 ml of DMEM-C. The cell suspension was transferred to a 15-ml centrifuge tube (Thermo Fisher Scientific, 339650), centrifuged for 5 min at 1500 rpm, and then the supernatant was discarded. Finally, 4–5 ml of DMEM-C was added, and cells were resuspended and seeded into a new T25 flask (Fig. 1). Repeat this enzymatic subculture protocol until the cells exhibit signs of senescence, such as morphological alterations and reduced cell division activity.

In this protocol, 200,000 cells were seeded for each subculture, and subsequent subculturing was performed 7–8 days later or once the cells reached a minimum of 80% confluence. Subculturing was repeated until passage 8 (P8). HDFs were identified in passages 3 (P3) and 8 (P8).

#### HDFs characterization

HDFs were characterized by identifying ACTIN and VIMENTIN proteins through immunofluorescence and *SERPINH1* gene expression using quantitative real-time PCR (RT-qPCR) at P3 and P8. ACTIN (red) and VIMENTIN (green) intensities were normalized to DAPI (blue) as the internal control, with results presented as mean ± SD from triplicate. The mean intensity was calculated using ImageJ mean grey value. *SERPINH1* expression was normalized to the reference gene GAPDH, and fold changes were calculated using the Livak (2^−ΔΔCt) method. Nuclear and cell areas were quantified using ImageJ’s Analyze Particle function.

Statistical analysis of ACTIN and VIMENTIN intensities between P3 and P8 was conducted using a paired t-test based on three sample subjects. The Wilcoxon signed-rank test was used to assess SERPINH1 fold changes between P3 and P8, also based on three sample subjects. Meanwhile, cell and nuclear area quantification was performed using the Mann-Whitney U test for non-parametric data, calculated from individual cells across all samples.

#### Immunofluorescence method

The cells were fixed with 4% paraformaldehyde for 10 min, permeabilized with 0.1% Triton™ X-100 for 15 min, and blocked with 1% BSA for 1 h at room temperature. The cells were labeled with VIMENTIN Mouse Monoclonal Antibody (Invitrogen, MA511883,) in 0.1% BSA with a dilution rate of 1:100 and incubated overnight at 4^o^C and then labeled with Goat anti-Mouse IgG (H + L) Superclonal™ Secondary Antibody, Alexa Fluor^®^ 488 conjugated (Invitrogen, A27034) at a dilution of 1:500 for 60 min at room temperature. Then, counterstained the samples with Rhodamine Phalloidin (Invitrogen, R415) at dilution 1:200 to stain F-ACTIN and DAPI (Invitrogen, 62248) to stain the nucleus. Lastly, the cell was mounted using SlowFade^®^ Gold Antifade Mountant. Cells were observed under Confocal Laser Scanning Microscope (LV1200, Olympus, 200x magnification).

#### Quantitative real-time PCR (RT-qPCR) method

The RT-qPCR was performed identify *SERPINH1* expression (forward 5’- GTGAGACCAAATTGAGCTAGGG-3’ and reverse 5’- TAGTTGGGAGAGGTTGGGATAG-3’). RNA was isolated from HDFs using Quick-RNA™ Miniprep Plus Kit (Zymo Research, R1057 ) [12]. RNA concentrations were measured by absorbance at 260 nm with a Biophotometer. A total of 2 µg of RNA was synthesized into cDNA using the SensiFAST cDNA Synthesis Kit (Meridian Bioscience, BIO-65053) with the RT-PCR thermocycler settings: 25 °C (10 min) for primer annealing; 42 °C (15 min) for reverse transcription; and 42 °C (15 min) for inactivation [13]. The synthesized cDNA samples were then analyzed via qPCR using the two-step PCR method with the SensiFAST SYBR No-ROX Kit (Meridian Bioscience, BIO-98005) with the qPCR settings: 95 °C (120s) for initial denaturation, followed by denaturation at 95 °C (5s) and annealing/elongation at 58 °C (20s) [14].

## Results

**Fig. 2:**
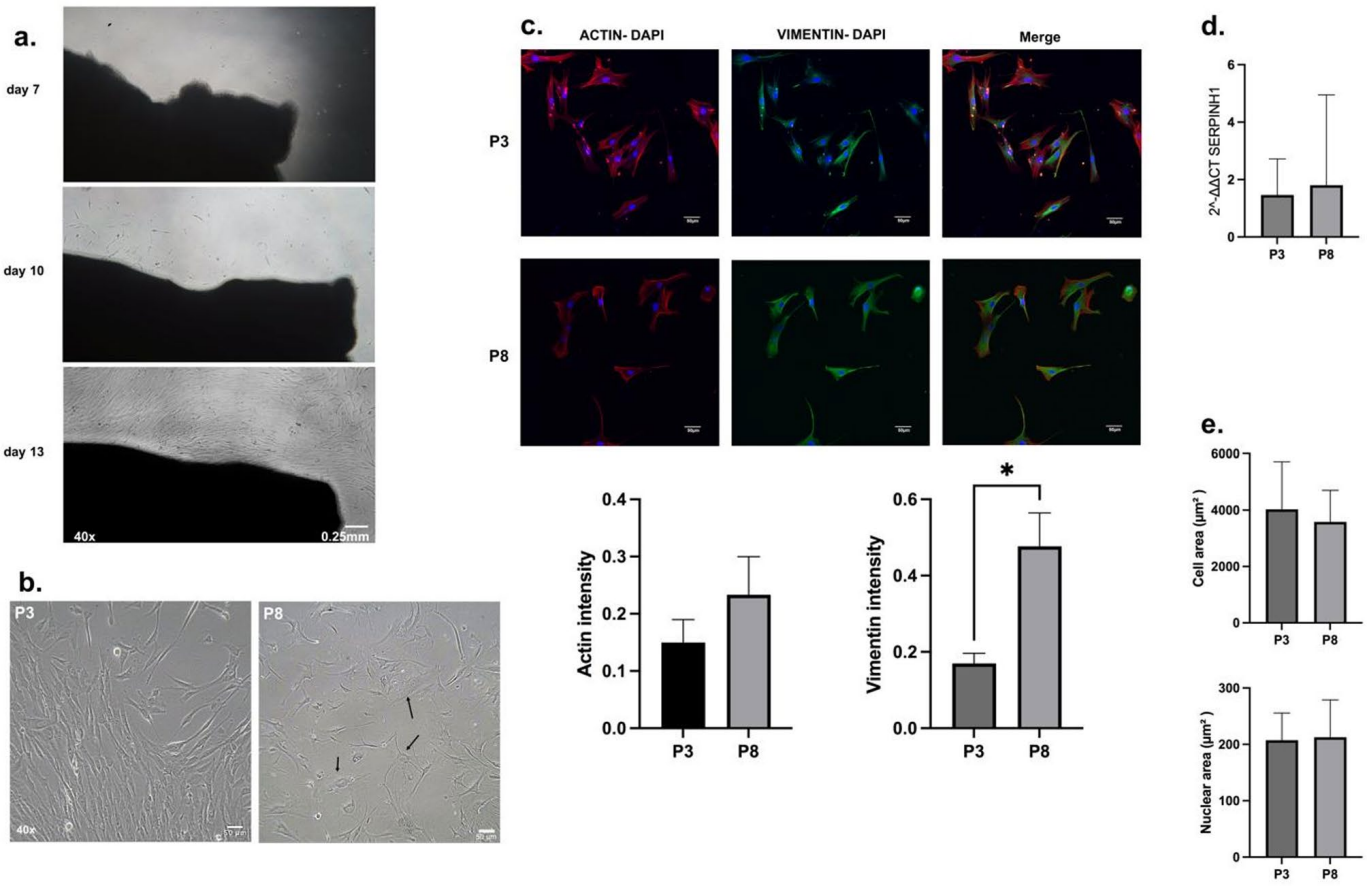
The morphology and characterization of HDFs. (**a**) The growth of HDFs from explant on days 7, 10, and 13. Spindle-shaped HDFs were initially detected on day ten after primary culture. (**b**) HDFs exhibit a thin, elongated, spindle-shaped morphology with tapered ends and an enlarged central cell diameter at early passage (P3). At late passage (P8), there is an increase in senescent cells, accompanied by an enlargement in cell size and nucleus and a transition from the thin, elongated spindle-shaped morphology to a flattened and irregular shape (black arrow). (**c**) HDFs were characterized by immunofluorescence, with ACTIN shown in red, VIMENTIN in green, and DAPI in blue. The images represent one field of view from a single sample. The lower panel shows intensity quantification from immunofluorescence. (**d**) *SERPINH1* expression in HDFs was characterized using RT-qPCR. (**e**) Quantification of cell and nuclear areas at P3 and P8. * = *p* < 0.05, indicates statistical significance

HDF growth typically becomes evident within approximately one week and reaches confluence within 2 to 6 weeks. Initially, HDFs appeared to grow from the edges of the explants (Fig. 2a). The expected outcomes of this protocol include HDFs exhibiting appropriate cell morphology and biomarker expression. HDFs at P3 displayed a thin, elongated spindle-shaped morphology with tapered ends and an enlarged central diameter (Fig. 2b), with a cell area of 4029.73 ± 1681.32 μm² and nuclear area 209.47 ± 48.44 μm² (Additional file 1, Table S2-S3). HDFs also expressed ACTIN 0.15 ± 0.04 and VIMENTIN 0.17 ± 0.02, which appeared as elongated fibers. The red-colored ACTIN was predominantly distributed at the cell periphery, extending towards the nucleus. At the same time, the green-colored VIMENTIN was primarily concentrated in the perinuclear region, extending towards the periphery (Fig. 2c). Moreover, *SERPINH1* expression exhibited a 1.46-fold change (Additional file 1, Table S4-S5).

Following repeated subculturing, HDFs at P8 showed morphological alterations and population heterogeneity (Fig. 2b), with a cell area of 3411.44 ± 1236.27 μm² and nuclear area 219.08 ± 62.7 μm² (Additional file 1, Table S2-S3). At P8, HDFs exhibited an increase in ACTIN (0.17 ± 0.02), VIMENTIN (0.48 ± 0.09), and *SERPINH1* (1.80-fold change) expression (Additional file 1, Table S4-S5).

Statistical analysis revealed a significant increase in VIMENTIN intensity at P8 compared to P3 (*p* = 0.027). Meanwhile, no significant changes were observed in ACTIN intensity (*p* = 0.054), *SERPINH1* fold changes (*p* = 0.4062), cell area (*p* = 0.581), or nuclear area (*p* = 0.894) between P3 and P8 (Fig. 2c-e).

## Discussion

Critical factors for successful HDF cultivation include meticulous sample selection, proper transportation, and thorough decontamination. As a primary culture, the donor’s physical condition may impact results. To prevent tissue degradation during transit, samples should be transported to the laboratory within one hour. According to Kabacik et al., samples can be stored in a tissue transport medium for up to 48 h [5].

As biological waste from genital organs, samples require thorough decontamination, especially cleaning residual urine attached to the tissue to mitigate the risk of contamination. Thorough washing of the sample with povidone-iodine and alcohol is crucial to eliminating microorganisms, as demonstrated by Ladke et al., while maintaining cell viability [15]. However, pay attention to the duration of washing, as an excessively prolonged washing period may lead to cell death. Alternatively, povidone-iodine utilization can be skipped, as demonstrated in a study by Nadalutti C et al. [2]. Kabacik et al. further noted that meticulous cleaning is not mandatory but enhances enzyme penetration [5].

We consider the absence of fibroblast growth within a 4-week to indicate experiment failure. Additional file 1, Table S6, presents troubleshooting encountered in primary culture when employing mechanical isolation and subculture methods, along with their corresponding causes and solutions.

This mechanical isolation protocol requires approximately 40–70 min for the culture process. Compared to this protocol, enzymatic isolation is time-consuming due to the time needed for enzyme digestion. For example, the enzyme liberase takes 16–20 h (or 3–4 h), while collagenase requires 1 h. Additionally, enzymatic isolation may seem more complex as it involves additional steps to halt the enzymatic process [2, 5]. In contrast, mechanical isolation reduces costs by eliminating enzyme use and extra steps (Table 1), making it a viable alternative to enzymatic isolation.

**Table 1:** Brief overview and comparison of alternate protocols

Step	Nadalutti C et al., 2020[8]	Kabacik S et al., 2022[11]	This Protocol
**Sample**	Human foreskin from young donor	Human foreskin from neonatal donor	Human foreskin from young donor (10 years old)
**Procedure**	Enzymatic isolation	Enzymatic isolation	Mechanical isolation
**Steps**:			
1. Decontamination	DPBS three times	Meticulous clean-up is not required	Povidone iodine, Alcohol 70%, DPBS three times. Discard the foreskin tip
2. Isolation	• Tissue mincing into 1–2 mm. • Enzymatic digestion with 200 µL of collagenase for 1 h. • Enzymatic digestion termination with 500 µL of complete medium. • Cell separation from tissue fragments using a cell strainer. • Sample centrifugation. • Sample washing with DPBS; then repeat the enzymatic digestion step.	• Tissue cutting into 5-6mm2 • Enzymatic digestion with 15 ml of liberase for **16–20 h (or alternatively**,** 3–4 h)** • Dermis separation mechanically	• Dermis separation mechanically • Tissue cutting into 1–2 mm.
3. Seeding	Seed 2 × 106 of the resuspended cell pellets to a gelatin-coated 35-mm petri dish with medium	• Seed 2–3 pieces of the dermis with the de-epidermized (pale) side face down in the 10 cm uncoated plate • Leave the explant without medium, then add medium after 15 min	• Seed six explants into a T-25 flask. • Leave the explants without medium, then add medium after 15 min.

We optimized repeated subculturing up to P8, as published in previous studies [16–18]. One of the primary challenges associated with repeated subculturing is the increased risk of contamination before reaching the desired passage. To mitigate this risk, flasks are recommended over well plates. As an alternative for studying natural aging, primary cultures from older donors could be considered [11]. However, the replicative senescence model remains more appropriate for time-series studies or investigations on preventive aging strategies [10]. 

Previous research has shown that HDFs have a spindle-shaped morphology, which is consistent with the findings in this experiment [10, 19]. Cellular senescence has been demonstrated to induce morphological alterations in cells, characterized by increased cell size and population heterogeneity [10, 19]. Makpol et al. found that senescent HDFs lose their fibroblastic shape and become flattened with increased cell size [20]. These findings are consistent with our study, where some P8 cells exhibited abnormal morphology under the phase contrast inverted microscope. (Fig. 2b). However, the mean cell area decreased (Fig. 2e). An increase in the nuclear area also accompanied the enlargement of the cell area during senescence [21–23], consistent with our results showing that the mean nuclear area increased from P3 to P8 (Fig. 2e).

The current protocol has successfully established HDFs with ACTIN and VIMENTIN expression through immunofluorescence and *SERPINH1* by RT-qPCR. ACTIN and VIMENTIN, as cytoskeletal elements, play critical roles in cell migration during wound healing [19]. ACTIN filaments are mainly at the cell periphery, connecting to the plasma membrane, while VIMENTIN is concentrated around the perinuclear region and anchors to desmosomes and hemidesmosomes [19, 24]. Our findings support this, as ACTIN and VIMENTIN localization influences cell biomechanics and organelle distribution [24, 25]. Previous research has revealed that senescence in HDFs is associated with increased expression of ACTIN and VIMENTIN, with VIMENTIN exhibiting a greater change compared to ACTIN. In this study, the change in VIMENTIN expression was significant, whereas the change in ACTIN was not. These findings align with the results of previous studies [19].

Additionally, *SERPINH1* plays a crucial role in stabilizing the collagen triple helix, the main component of the extracellular matrix in the dermis [26]. Previous research has revealed that senescence in fibroblast leads to decreased SERPINH1 expression, resulting in reduced collagen expression [26, 27]. However our findings showed that the expression of SERPINH1 has tendency to increase at P8, even though the difference was not significant. It indicates that the *SERPINH1* gene remains suitable for characterizing HDFs up to P8.

Furthermore, previous studies have reported that increased collagen levels can lead to a reduction in cell area [28]. The decrease in cell area observed in our study aligns with the increase in SERPINH1 expression, which is known to influence collagen production [26]. Hence, these results suggest that P8 was still undergoing the aging process but had not yet reached the final stage of cellular senescence. This aging process was evidenced by a significant increase in VIMENTIN expression, a non-significant upward trend in ACTIN levels and nuclear area, and the presence of morphological abnormalities at P8.

This protocol utilizes a cost-effective and straightforward mechanical isolation method utilizing the foreskin, which is considered biological waste. HDFs can endure subculturing up to P8, making it applicable for studies on intrinsic skin aging, particularly under limited funding and resources.

### Limitations

This protocol can be challenging, as separating the epidermis and adipose tissue from the dermis is easier with enzymatic methods. Mechanical isolation is less effective for adult foreskin donors due to large fat deposits. Maintaining cell viability after multiple subculturing steps is complex, and results may vary regarding the time required for cell growth and confluence, especially in later passages.

## Electronic supplementary material

Below is the link to the electronic supplementary material.

Supplementary Material 1

## Data Availability

All data generated or analyzed during this study are included in this published article [and its supplementary information files].

## Abbreviations

HDFs: Human dermal fibroblasts
RT-qPCR: reverse transcription-quantitative polymerase chain reaction
BSL: Biosafety Level
DMEM-C: Dulbecco’s Modified Eagle Complete medium
DPBS: Dulbecco’s Phosphate Buffered Saline
FBS: Fetal Bovine Serum
