# Supplementary material for: Efficient and simple protocol for mechanical isolation of foreskin-derived primary human dermal fibroblasts for replicative senescence studies
Source: BMC Res Notes. 2025 Jul 18;18:311. doi: 10.1186/s13104-025-07373-2 (PMC12275237; doi:10.1186/s13104-025-07373-2)
Supplement: Supplementary file 1 — Supplementary Material 1 [file 13104_2025_7373_MOESM1_ESM.docx]

# Table S1: Reagents and solutions ingredients

| **Reagents and Solutions** | **Ingredients** | **Volume (Concentration)** |
| --- | --- | --- |
| Transport medium (25 ml) | Antibiotic Antimycotic Solution 100x, Stabilized (Sigma-Aldrich, A5955 ) | 0.75 ml (3% v/v) |
|  | Fetal Bovine Serum Honduras Origin Sterile Filtered (Sigma-Aldrich, F7524), heat inactivated (55^o^C, 30 minutes) | 2.5 ml (10% v/v) |
|  | Dulbecco’s Modified Eagle Medium 1X with 1 g/L D-Glucose, L-Glutamine, 110 mg/L Sodium Pyruvate (Gibco, 11885-084) | 21.75 ml |
|  | *Mix well in 50 ml Conical Centrifuge Tube (Thermo scientific, 339652)* | |
| DMEM-C (50 ml) | Antibiotic Antimycotic Solution 100x, Stabilized (Sigma-Aldrich, USA, A5955) | 0.5 ml (1% v/v) |
|  | Fetal Bovine Serum Honduras Origin Sterile Filtered (Sigma-Aldrich, F7524), heat inactivated (55^o^C, 30 minutes) | 5 ml (10% v/v) |
|  | Dulbecco’s Modified Eagle Medium 1X with 1 g/L D-Glucose, L-Glutamine, 110 mg/L Sodium Pyruvate (Gibco, 11885-084) | 44.5 ml |
|  | *Mix well in 50 ml Conical Centrifuge Tube (Thermo scientific, 339652)* | |
| DPBS-1X (50 ml) | Antibiotic Antimycotic Solution 100x, Stabilized (Sigma-Aldrich, A5955) | 0.5 ml (1% v/v) |
|  | Dulbecco’s Phospate Buffered Saline without calcium, without magnesium (Sigma-Aldrich, 59331C) | 5 ml (1X) |
|  | Aquades | 44.5 ml |
|  | *Mix well in 50 ml Conical Centrifuge Tube (Thermo scientific, 339652)* | |
| Trypsin 0.05% (15 ML) | Trypsin – EDTA Solution (Sigma-Aldrich, T4049) | 3 ml (0.05% v/v) |
|  | DPBS-1X | 12 ml |
|  | *Mix well in 15 ml Conical Centrifuge Tube (Thermo scientific,* 339650*)* | |

# Table S2: Cell area

| P3 | CELL AREA |
| --- | --- |
| 1 | 4027.102 |
| 2 | 5492.482 |
| 3 | 3698.469 |
| 4 | 6715.428 |
| 5 | 3919.353 |
| 6 | 3674.225 |
| 7 | 4607.597 |
| 8 | 4315.329 |
| 9 | 5095.159 |
| 10 | 2797.422 |
| 11 | 2649.267 |
| 12 | 9403.754 |
| 13 | 3391.385 |
| 14 | 1926.005 |
| 15 | 3969.187 |
| 16 | 3710.59 |
| 17 | 1643.165 |
| 18 | 4227.783 |
| 19 | 3061.405 |
| 20 | 4722.08 |
| 21 | 3439.872 |
| 22 | 2167.093 |
| AVG | 4029.734182 |
| SD | 1681.324354 |
|  |  |
|  |  |
| P8 | CELL AREA |
| 1 | 1935.714 |
| 2 | 3207.412 |
| 3 | 4030.427 |
| 4 | 2531.957 |
| 5 | 3923.761 |
| 6 | 4429.352 |
| 7 | 3362.979 |
| 8 | 2868.255 |
| 9 | 4302.382 |
| 10 | 4100.775 |
| 11 | 5995.024 |
| 12 | 2291.172 |
| AVG | 3581.6 |
| SD | 1121.02 |

# Table S3: Nucleus area

| P3 | NUCLEUS AREA |
| --- | --- |
| 1 | 184.038 |
| 2 | 227.898 |
| 3 | 190.918 |
| 4 | 270.897 |
| 5 | 215.858 |
| 6 | 188.338 |
| 7 | 235.638 |
| 8 | 230.478 |
| 9 | 139.319 |
| 10 | 239.938 |
| 11 | 180.598 |
| 12 | 197.798 |
| 13 | 202.098 |
| 14 | 170.278 |
| 15 | 157.378 |
| 16 | 135.879 |
| 17 | 216.718 |
| 18 | 360.336 |
| 19 | 237.358 |
| 20 | 214.998 |
| 21 | 202.098 |
| 22 | 162.538 |
| AVG | **209.469** |
| ST DEV | **48.442** |
|  |  |
| P8 | NUCLEUS AREA |
| 1 | 267.457 |
| 2 | 225.318 |
| 3 | 189.198 |
| 4 | 215.858 |
| 5 | 276.917 |
| 6 | 178.878 |
| 7 | 282.937 |
| 8 | 190.058 |
| 9 | 347.436 |
| 10 | 134.159 |
| 11 | 147.918 |
| 12 | 214.138 |
| 13 | 318.197 |
| 14 | 188.338 |
| 15 | 175.438 |
| 16 | 153.078 |
| 17 | 108.359 |
| AVG | **219.083** |
| ST DEV | **62.700** |

# Table S4: ACTIN & VIMENTIN intensity normalize to DAPI

|  | ACTIN P3 | ACTIN P8 | VIMENTIN P3 | VIMENTIN P8 |
| --- | --- | --- | --- | --- |
| Slide 1 | 0.15 | 0.20 | 0.15 | 0.55 |
| Slide 2 | 0.11 | 0.19 | 0.20 | 0.50 |
| Slide 3 | 0.19 | 0.31 | 0.16 | 0.38 |
| mean | 0.15 | 0.23 | 0.17 | 0.48 |
| SD | 0.04 | 0.07 | 0.02 | 0.09 |

# Table S5: SERPIN H1 GENE EXPRESSION

|  | CT SerpinH1 | CT SerpinH1 | CT GAPDH | ΔCT 1 | ΔCT 2 | Avg ΔCT | 2^-ΔΔCT 1 | 2^-ΔΔCT 2 |
| --- | --- | --- | --- | --- | --- | --- | --- | --- |
| P3-A | 17.23 | 14.11 | 17.31 | -0.08 | -3.2 | -1.64 | 0.34 | 2.95 |
| P8-A | 19.2 | 19.05 | 17.96 | 1.24 | 1.09 |  | 0.14 | 0.15 |
| P3-B | 19.17 | 15.93 | 16.455 | 2.715 | -0.525 | 1.10 | 0.33 | 3.07 |
| P8-B | 14.51 | 17.08 | 16.44 | -1.93 | 0.64 |  | 8.14 | 1.37 |
| P3-C | 15.89 | 15.07 | 13.115 | 2.775 | 1.955 | 2.37 | 0.75 | 1.33 |
| P8-C | 16.49 | 14.66 | 11.965 | 4.525 | 2.695 |  | 0.22 | 0.80 |
| Average P3 = | | | | | | | 1.46 | |
| Average P8 = | | | | | | | 1.80 | |
| p-*value* | | | | | | | 0.4062 | |

# Table S6: Troubleshooting and the solutions

| **Problems** | **Possible causes** | **Solutions** |
| --- | --- | --- |
| Culture | | |
| Contamination | Reagent contamination | Replace the current reagent with new ones, or filter the reagent with a syringe-filter |
|  | Unsterilized tools | Autoclave all the tools. Use alcohol 70% on the tools before putting in BSC.  Clean the incubator  Using flasks is more recommended than well-plates |
|  | Inadequate sample decontamination | Review the sample decontamination procedure. Thoroughly remove remnants, such as urine, from the foreskin tip |
| HDFs are not growing | Inappropriate medium composition | Evaluate the current DMEM-C composition. Consider increasing Fetal Bovine Serum (FBS) concentration to 12-15% |
|  | Incorrect incubator settings | Adjust the incubator setting. Check the accuracy of the incubator sensors. Verify the availability of CO2 levels in the incubator. |
|  | Explants failure to adhere | Let the explants sit for 15 minutes without any medium when seeding the explant.  Handle flask transfer and medium replacement with care to prevent any disturbance to the explant. |
|  | Significant cell death due to prolonged transportation and prolonged use of povidone-iodine and alcohol 70%. | Expedite the transportation and cleansing procedures. |
| Difficulty separating epidermis and adipose tissue from dermis | Inappropriate surgical set | Utilize sharp scissors and a surgical blade. |
|  | Using sample from adult donor | Select samples from younger donor. Generally, the younger the age, the easier it is to separate the epidermis and adipose tissue from dermis. |
| Keratinocytes or adipocytes growth | Inappropriate epidermis and adipose tissue cleaning | Reevaluate the mechanical isolation procedures. |
|  | Using sample from adult donor | Adult skin samples often contain large fat deposits that are hard to clean. Select samples from younger donors. |
|  |  | The adipocytes and keratinocytes will commonly disappear after subculture because of the unsuitable growth medium. |
| Subculture | | |
| Cells not adhering after subculture | Trypsin concentration too high, prolonged incubation with trypsin | Review the subculture process. |
|  | Inadequate medium composition for trypsin inactivation | Use complete medium solutions with volume two times trypsin volume |
| Contamination | Reagent contamination | Replace the current reagent with new ones, or filter the reagent with a syringe-filter |
|  | Unsterilized tools | Autoclave all the tools. Use alcohol 70% on the tools before putting in BSC.  Clean the incubator  Using flasks is more recommended than well-plates |
| Cells exhibit no apparent growth | Replicative senescence has occurred | Perform assessment of replicative senescence  Subsequent subculturing can be conducted in containers with a smaller surface area (e.g., well plates) |
